# Role of p53 in Cisplatin-Induced Myotube Atrophy

**DOI:** 10.3390/ijms24119176

**Published:** 2023-05-24

**Authors:** Chinami Matsumoto, Hitomi Sekine, Nana Zhang, Sachiko Mogami, Naoki Fujitsuka, Hiroshi Takeda

**Affiliations:** 1 Tsumura Kampo Research Laboratories, Tsumura & Co., 3586 Yoshiwara, Ami-machi, Inashiki-gun 300-1192, Japan; 2 Gastroenterology, Tokeidai Memorial Hospital, 2-3 North-1, East 1, Chuo-ku, Sapporo 060-0031, Japan; 3 Faculty of Pharmaceutical Sciences, Hokkaido University, Kita 12, Nishi 6, Kita-ku, Sapporo 060-0812, Japan

**Keywords:** p53, cisplatin, C2C12 myotube, atrophy, pifithrin-alpha

## Abstract

Chemotherapy-induced sarcopenia is an unfavorable prognostic factor implicated in the development of postoperative complications and reduces the quality of life of patients with cancer. Skeletal muscle loss due to cisplatin use is caused by mitochondrial dysfunction and activation of muscle-specific ubiquitin ligases Atrogin-1 and muscle RING finger 1 (MuRF1). Although animal studies suggest the involvement of p53 in age-, immobility-, and denervation-related muscle atrophy, the association between cisplatin-induced atrophy and p53 remains unknown. Herein, we investigated the effect of a p53-specific inhibitor, pifithrin-alpha (PFT-α), on cisplatin-induced atrophy in C2C12 myotubes. Cisplatin increased the protein levels of p53, phosphorylated p53, and upregulated the mRNA expression of p53 target genes *PUMA* and *p21* in C2C12 myotubes. PFT-α ameliorated the increase in intracellular reactive oxygen species production and mitochondrial dysfunction, and also reduced the cisplatin-induced increase in the *Bax/Bcl-2* ratio. Although PFT-α also reduced the cisplatin-induced increase in *MuRF1* and *Atrogin-1* gene expression, it did not ameliorate the decrease in myosin heavy chain mRNA and protein levels and muscle-specific actin and myoglobin protein levels. We conclude that cisplatin increases muscle degradation in C2C12 myotubes in a p53-dependent manner, but p53 has minimal involvement in the reduction of muscle protein synthesis.

## 1. Introduction

Loss of skeletal muscle (sarcopenia) has a negative impact on the quality of life, disease course, and life expectancy of the affected patients [1,2,3]. The condition is classified as primary sarcopenia when the muscle loss is associated with aging, whereas it is classified as secondary sarcopenia when the muscle loss is associated with reduced activity (disuse atrophy), malnutrition, organ failure, invasion, tumors, and other diseases [2,3]. In particular, cachexia, malnutrition (from reduced food intake), and low activity levels can render patients with cancer vulnerable to developing secondary sarcopenia, which increases their postoperative complications and decreases their long-term survival ability [4]. Sarcopenia also reduces the efficacy of cancer chemotherapy and increases the frequency of adverse events. Therefore, this condition is an important problem affecting the long-term survival of patients [5,6,7]. Although it is known that chemotherapy-associated malnutrition progresses skeletal muscle loss in patients with cancer, it has recently been shown that anticancer drugs per se can cause secondary sarcopenia and a cachexia-like condition [8,9,10].

Cisplatin, which was first developed in the 1970s, is currently a standard chemotherapy agent used for the treatment of a broad range of cancers, including those of the gastrointestinal tract, lungs, head, neck, testicles, bladder, ovaries, and cervix. Previous studies have demonstrated that cisplatin causes skeletal muscle atrophy by suppressing the synthesis and increasing the degradation of muscle proteins [11,12,13,14]. Although the exact mechanisms through which cisplatin causes skeletal muscle atrophy remain unclear, mitochondrial damage and overproduction of reactive oxygen species (ROS) have been implicated [15]. We have previously reported that cisplatin-induced mitochondrial damage and elevated ROS levels are involved in C2C12 myotube atrophy [16]. The Bax/Bcl-2 ratio is positively correlated with mitochondrial outer membrane permeabilization and the induction of apoptosis [17]. Additionally, an increase in the Bax/Bcl-2 ratio is associated with muscle atrophy induced by anticancer drugs [18,19]. In our previous study, we observed that cisplatin-induced mitochondrial ROS production raised the ratio of BCL2-associated X protein (*Bax*) to B cell leukemia/lymphoma 2 (*Bcl-2*), thereby causing mitochondrial dysfunction. The mitochondrial damage resulted in sustained ROS production, which in turn, increased the gene expression of *Atrogin-1* (a muscle atrophy factor) and decreased that of the myosin heavy chain (MyHC) protein. These effects were inhibited by mitoquinone mesylate (MitoQ), an antioxidant that targets mitochondria. Cisplatin treatment also decreased mitochondrial respiration and ATP production by the glycolytic system in myotubes; however, these effects were not implicated in myotube atrophy [16]. Activation of p53 is known to increase the *Bax/Bcl-2* ratio and suppress the glycolytic system [20,21,22]; it has occurred during cisplatin-induced kidney and inner ear cell damage [23,24,25].

The above-mentioned findings suggest that p53 activation may be critical for the progression of cisplatin-induced myotube atrophy. However, this aspect was not investigated in our previous report. Therefore, the aim of this study was to elucidate the involvement of p53 in cisplatin-induced C2C12 myotube atrophy using pifithrin-alpha (PFT-α) [26,27], a p53-specific inhibitor, to study the relationships between p53, mitochondrial function, and myotube atrophy. Here, we demonstrated that cisplatin activated p53 and increased the *Bax/Bcl-2* ratio and mitochondrial dysfunction, resulting in increased muscle proteolysis in C2C12 myotubes. These results suggest that p53 may be involved in part of the mechanisms involved in cisplatin muscle atrophy.

## 2. Results

### 2.1. Cisplatin Induced p53 Activation in C2C12 Myotubes

**Figure 1:**
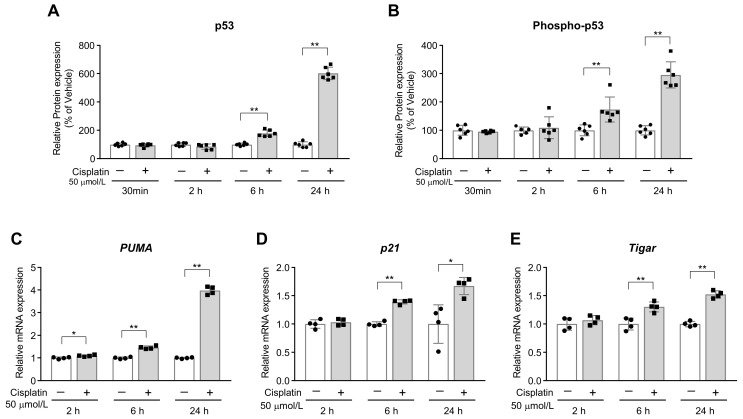
Expression of p53 in cisplatin-treated C2C12 myotubes. (**A**,**B**) Protein levels of (**A**) p53 and (**B**) phospho-p53 (*n* = 6). (**C**–**E**) The mRNA expression levels of p53 target genes, namely (**C**) p53 up-regulated modulator of apoptosis (*PUMA*), (**D**) *p21* and (**E**) *Tigar* (*n* = 4). Data are shown as the mean ± SD. * *p* < 0.05, ** *p* < 0.01, Student’s or Welch’s *t* test.

To begin with, we investigated whether cisplatin activated p53 in C2C12 myotubes. At 6 h after treatment, the levels of p53 and phosphorylated p53 (phospho-p53) were higher in the cisplatin-treated cells than those in the vehicle-treated cells, and the difference was even more pronounced after 24 h of treatment (Figure 1A,B). Similarly, the expression levels of the p53 target genes, viz. p53 upregulated modulator of apoptosis (*PUMA*), *p21*, and TP53-induced glycolysis and apoptosis regulator (*Tigar*), were increased by cisplatin treatment (Figure 1C–E). These findings showed that p53 was activated by cisplatin in C2C12 cells.

**Figure 2:**
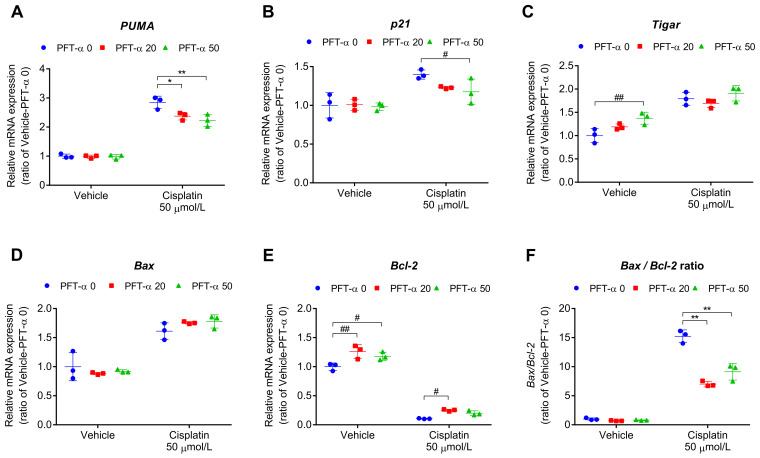
Effect of the p53 inhibitor PFT-α (20 or 50 μmol/L) on the expression of p53 target and mitochondrial apoptotic regulator genes in C2C12 myotubes treated with cisplatin for 24 h. (**A**) *PUMA*, (**F**) *Bax/Bcl-2* ratio; two-way ANOVA revealed a significant effect of cisplatin (*p* < 0.01) and cisplatin × PFT-α (*p* < 0.01). * *p*  <  0.05, ** *p*  <  0.01, Tukey’s multiple-comparisons post hoc test. (**B**) *p21*, (**C**) *Tigar*, (**D**) *Bax*, (**E**) *Bcl-2*; two-way ANOVA revealed a significant effect of cisplatin (*p* < 0.01). # *p*  <  0.05, ## *p*  <  0.01, Dunnett’s post hoc test. Data are shown as the mean ± SD (*n* = 3).

The p53 inhibitor PFT-α reduced the cisplatin-induced upregulated expression of *PUMA* and *p21* (Figure 2A,B). However, PFT-α had no effect on *Tigar* expression levels (Figure 2C).

Cisplatin treatment for 24 h increased *Bax* expression by 1.6-fold (Figure 2D) and reduced the expression of *Bcl-2* to 1/10th of its original level (Figure 2E), which effectively led to a 15-fold increase in the *Bax/Bxl-2* ratio (Figure 2F). Although PFT-α had no effect on *Bax* expression (Figure 2D), it significantly increased the *Bcl-2* expression level (Figure 2E), thereby causing a marked decrease in the *Bax/Bcl-2* ratio (Figure 2F).

### 2.2. Involvement of p53 in Cisplatin-Induced Mitochondrial Dysfunction

**Figure 3:**
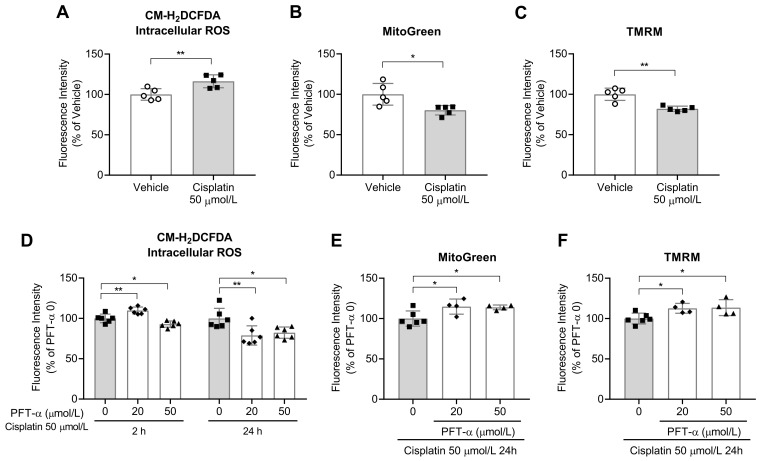
Effect of PFT-α on mitochondrial function in cisplatin-treated C2C12 myotubes. (**A**,**D**) Intracellular ROS levels (*n* = 5–6). (**B**,**E**) Mitochondrial mass and (**C**,**F**) Mitochondrial membrane potential (*n* = 4–6). Data are shown as the mean ± SD. * *p* < 0.05, ** *p* < 0.01, Student’s *t* test (**A**–**C**) or Dunnett’s test (**D**–**F**).

In our previous study, we noted an increase in intracellular levels of ROS 24 h after cisplatin treatment [16]. However, in this study, the increase in ROS levels in the treated cells occurred just after 2 h of treatment (Figure 3A). PFT-α (50 µmol/L) reduced the intracellular ROS levels at both 2 and 24 h after cisplatin treatment (Figure 3D). After 24 h, the mitochondrial mass and membrane potential in the cisplatin-treated cells were both found to be lower than those in vehicle-treated cells (Figure 3B,C); however, the levels were increased upon the addition of PFT-α (Figure 3E,F). These findings suggested that p53 was potentially involved in cisplatin-induced ROS production and mitochondrial damage.

**Figure 4:**
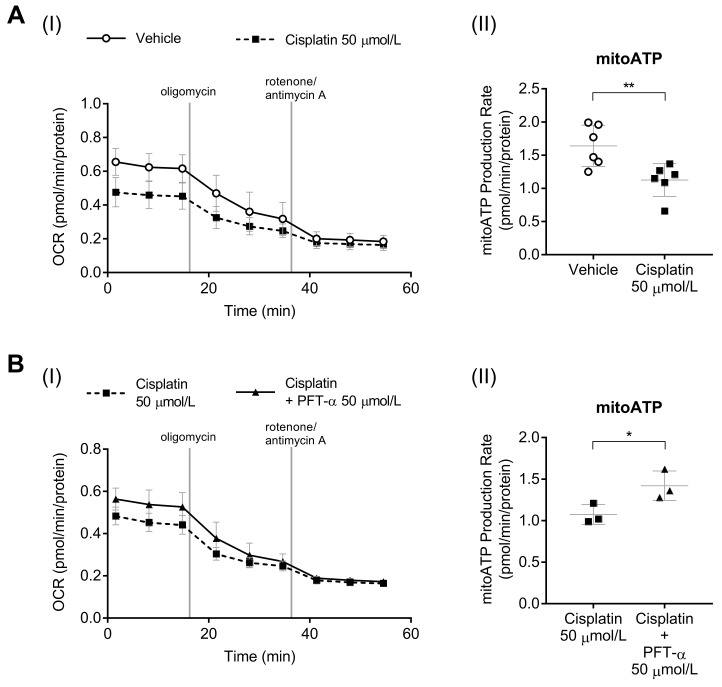
Effect of PFT-α on mitochondrial respiration and mitochondrial ATP production. Real-time measurements of oxygen consumption rate (OCR) in C2C12 myotubes treated with vehicle, cisplatin, or cisplatin + PFT-α for 24 h. (**A**) Cisplatin-induced mitochondrial hypofunction. (**B**) Effect of PFT-α on mitochondrial hypofunction. The data show the (**I**) mitochondrial respiration (OCR) and (**II**) mitochondrial ATP (mitoATP) production rate. Data are presented as the mean ± SD (*n* = 3–6). * *p* < 0.05, ** *p* < 0.01, Student’s *t* test.

We next investigated the involvement of p53 in mitochondrial function. This was estimated by using the oxygen consumption rate (OCR) as an indicator of mitochondrial oxidative phosphorylation (Figure 4(AI,BI)) and by determining the adenosine triphosphate (ATP) production rate via mitochondrial respiration. Cisplatin treatment significantly reduced the rate of mitochondrial ATP production (Figure 4(AII)), whereas PFT-α treatment increased this rate by approximately 1.3-fold compared with that upon treatment with cisplatin alone (Figure 4(BII)).

### 2.3. Involvement of p53 in Cisplatin-Induced Suppression of the Glycolytic System

**Figure 5:**
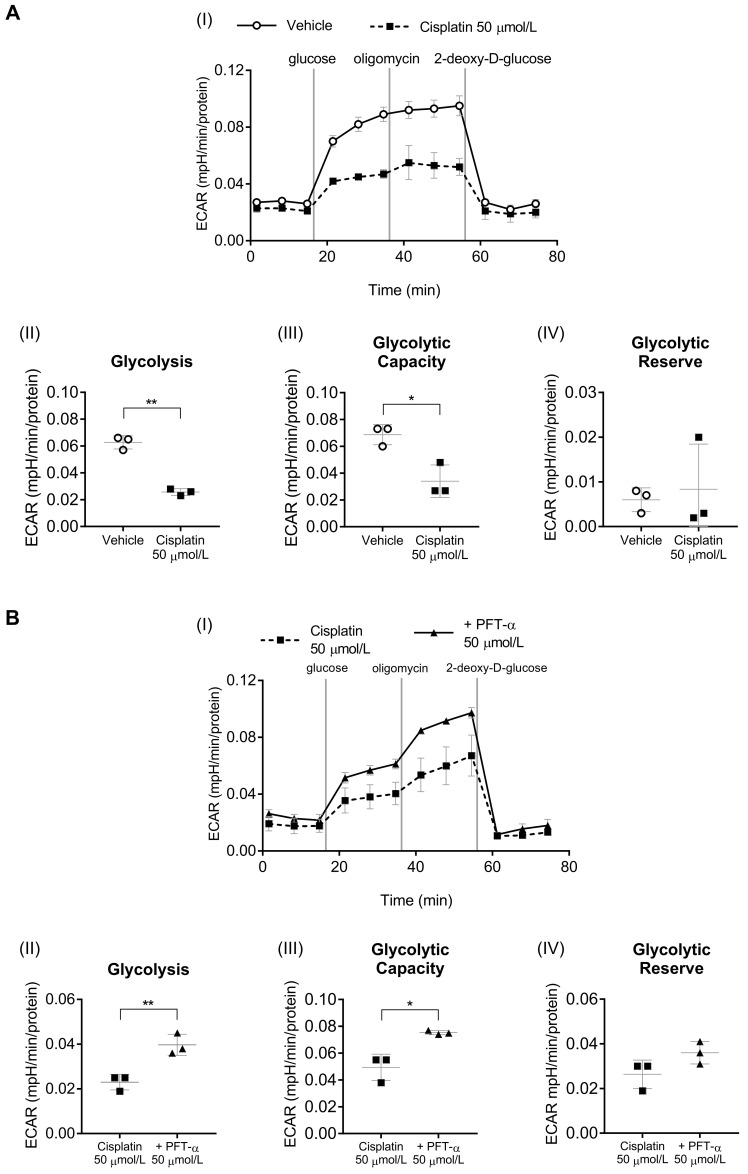
Glycolytic flux in C2C12 myotubes. (**A**) Impairment of glycolytic function by cisplatin. (**B**) Effect of PFT-α on glycolytic function. (**I**) Time-course data showing extracellular acidification rate (ECAR) levels after treatment with glucose, the ATP synthesis inhibitor oligomycin, and the glycolysis inhibitor 2-deoxy-D-glucose. Glycolysis levels (**II**), glycolytic capacity (**III**), and glycolytic reserve (**IV**) were estimated using ECAR analysis. Data are presented as the mean ± SD (*n* = 3). * *p* < 0.05, ** *p* < 0.01, Student’s or Welch’s *t* test.

As an indicator of glycolysis, the extracellular acidification rate (ECAR) was measured (Figure 5(AI,BI)). Cisplatin significantly decreased the levels of glycolysis (Figure 5(AII)) and glycolytic capacity (Figure 5(AIII)) of C2C12 myotubes but had no clear impact on their glycolytic reserve (Figure 5(AIV)). Adding PFT-α to the cisplatin-treated cells caused a significant increase in their glycolysis (Figure 5(BII)) and glycolytic capacity (Figure 5(BIII)). Moreover, it showed a tendency to increase the glycolytic reserve (Figure 5(BIV)). These findings suggested the involvement of p53 in cisplatin-induced reduction of myotube glycolytic capacity.

### 2.4. Involvement of p53 in Cisplatin-Induced Reduction of Muscle Atrophy-Related Gene Expression

**Figure 6:**
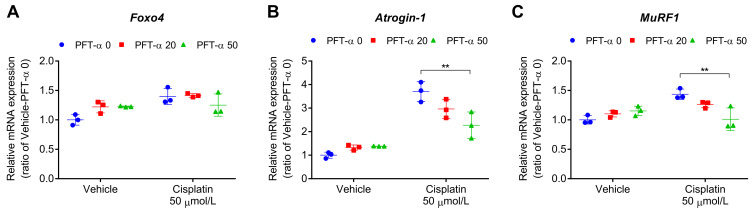
Effect of PFT-α (20 or 50 μmol/L) on the expression of muscle atrophy-related genes in C2C12 myotubes treated with cisplatin for 24 h. (**A**) *Foxo4*, (**B**) *Atrogin-1*, (**C**) *MuRF1*; two-way ANOVA revealed a significant effect of cisplatin (*p* < 0.01) and cisplatin × PFT-α (*p* < 0.05). ** *p*  <  0.01, Tukey’s multiple-comparisons post hoc test. Data are shown as the mean ± SD (*n* = 3).

Next, we investigated the impact of PFT-α on specific atrogenes, namely *MuRF1*, *Atrogin-1*, and forkhead box O (Foxo) family genes (*Foxo1*, *Foxo3a*, and *Foxo4*). Among the Foxo family genes, we specifically focused on Foxo4 due to its strong association with cisplatin-induced C2C12 myotube atrophy as demonstrated in our previous study [16]. PFT-α caused a marginal (statistically non-significant) decrease in the cisplatin-induced increase in the expression of *Foxo4* gene (Figure 6A), and significantly reduced the cisplatin-induced increase in *Atrogin-1* and *MuRF1* gene expression (Figure 6B,C). These findings indicated the involvement of p53 in the cisplatin-induced activation of the ubiquitin–proteasome system.

### 2.5. Involvement of p53 in Cisplatin-Induced Reduction in Muscle Protein Expression

**Figure 7:**
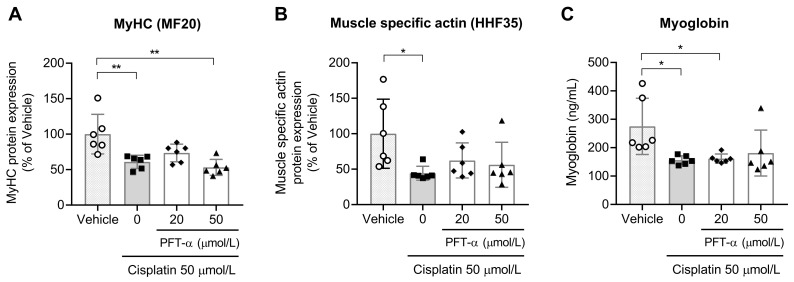
Effect of PFT-α on cisplatin-induced muscle-related protein expression in C2C12 myotubes treated with cisplatin for 24 h. (**A**–**C**) Evaluation of the protein levels of (**A**) MyHC, (**B**) muscle specific actin, and (**C**) myoglobin in C2C12 myotubes (*n* = 6). Data are shown as the mean ± SD (*n* = 6). * *p* < 0.05, ** *p* < 0.01, Tukey’s test.

When considering muscle protein expression, cisplatin treatment caused a significant reduction in the levels of MyHC, muscle-specific actin, and myoglobin; PFT-α failed to improve the levels of these three proteins (Figure 7, Supplementary Figure S1).

### 2.6. Involvement of p53 in Cisplatin-Induced Reduction in the MyHC Isoforms and Myogenic Regulatory Factor Gene Expression

Given that PFT-α could suppress the degradation of muscle proteins but did not restore their expression levels, we investigated its effect on muscle protein synthesis. In rodent skeletal muscle fibers, the following four different MyHC isoforms exist: a slow oxidative type (MyHC I), a fast oxidative type (MyHC IIA), an intermediate type (MyHC IIX), and a fast glycolytic type (MyHC IIB); these are encoded by *Myh7*, *Myh2*, *Myh1*, and *Myh4* genes, respectively [28]. We compared PFT-α with MitoQ (an antioxidant that targets mitochondria) in terms of their effects on the gene expression of MyHC isoforms.

**Figure 8:**
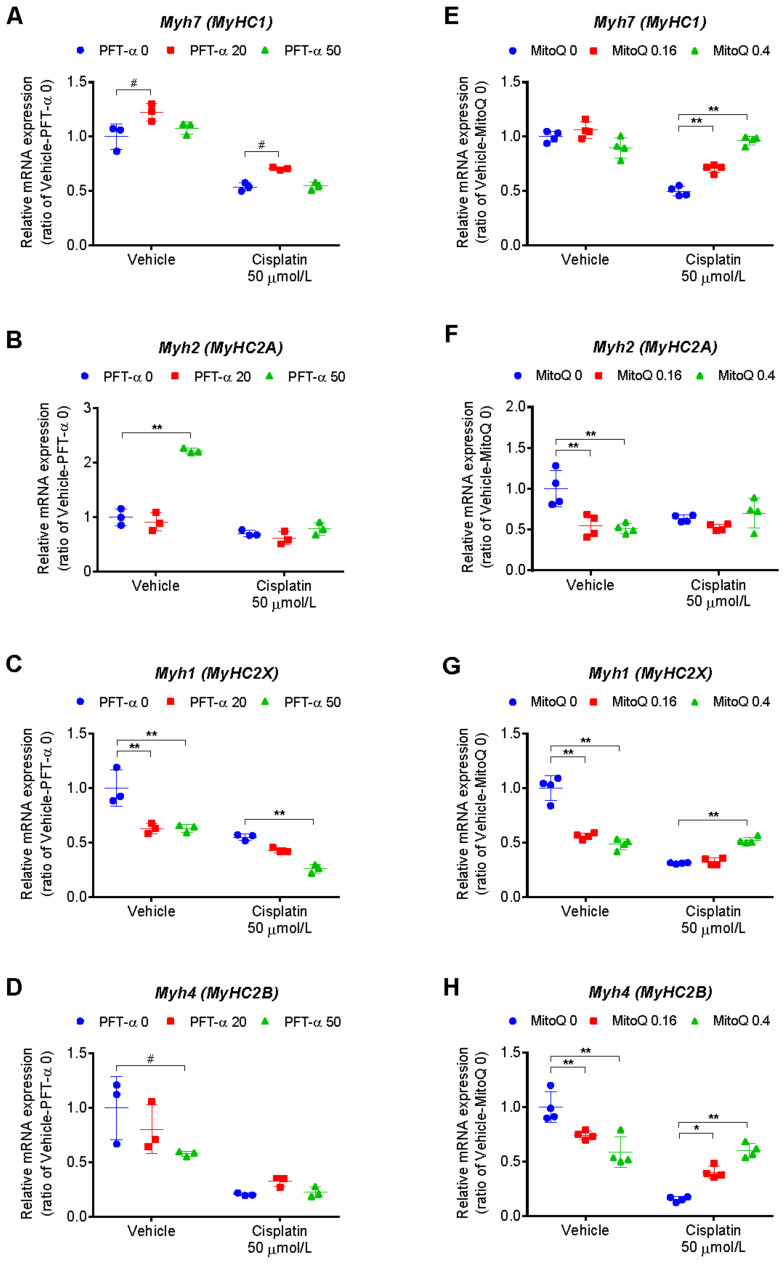
Effects of PFT-α and the mitochondria-specific antioxidant MitoQ on myosin heavy chain (MyHC) isoform gene expression. (**A**–**D**) The mRNA expression levels in C2C12 myotubes treated with cisplatin for 24 h with 20 or 50 μmol/L PFT-α or (**E**–**H**) 0.16 or 0.4 µmol/L MitoQ. (**A**) *Myh7* and (**D**) *Myh4*; two-way ANOVA revealed a significant effect of cisplatin (*p* < 0.01). # *p*  <  0.05, Dunnett’s post hoc test. (**B**) *Myh2*, (**C**,**G**) *Myh1*, (**E**) *Myh7*, and (**H**) *Myh4*; two-way ANOVA revealed a significant effect of cisplatin (*p* < 0.01) and cisplatin × PFT-α or MitoQ (*p* < 0.05). (**F**) *Myh2*; two-way ANOVA revealed a significant effect of cisplatin × MitoQ (*p* < 0.01). * *p*  <  0.05, ** *p*  <  0.01, Tukey’s multiple-comparisons post hoc test. Data are shown as the mean ± SD (*n* = 3–4).

Cisplatin significantly reduced the expression of *Myh7*, *Myh2*, *Myh1*, and *Myh4* genes in C2C12 myotubes (Figure 8), whereas MitoQ significantly improved the expression of all isoforms except for fast-type *Myh2* (Figure 8E–H). In contrast, PFT-α significantly improved the expression levels of fast-type *Myh7* at 20 µmol/L, had no effect on *Myh7* at 50 µmol/L (Figure 8A), and had no effect on any of the other isoforms at either concentration (Figure 8B–D). Additionally, cisplatin reduced the mRNA expression levels of myogenic differentiation 1 (*MyoD1*), myogenin, and insulin-like growth factor 1 (*Igf1*) genes, and this effect could not be reversed by PFT-α treatment. In contrast, MitoQ significantly ameliorated the cisplatin-induced reduction in myogenin mRNA levels (Supplementary Figure S2).

## 3. Discussion

In this study, we sought to elucidate the role of p53 in cisplatin-induced C2C12 myotube atrophy. Cisplatin increased the protein levels of p53 and phospho-p53 as well as the mRNA levels of p53 target genes in C2C12 cells. PFT-α, an inhibitor of p53 transcriptional activity, reduced mitochondrial damage, ROS production, the cisplatin-induced increase in the *Bax/Bcl-2* ratio, and activation of the muscle protein degradation system. This indicates a direct involvement of increased p53 transcriptional activity in these processes. Conversely, PFT-α did not ameliorate the cisplatin-induced decrease in the MyHC mRNA and protein levels as well as muscle-specific actin and myoglobulin protein levels. Based on these findings, we concluded that p53 exerts dissimilar effects in the degradation and synthesis of muscle proteins; it plays an important role in cisplatin-activated muscle protein degradation but has only a minor role in cisplatin-induced suppression of protein synthesis.

The involvement of p53 in muscle atrophy has been widely studied; however, the conclusions of these studies have been contradictory. For example, some studies have reported enhanced *p53* mRNA expression in the skeletal muscle of elderly people and aged animals [29,30,31,32], as well as enhanced p53 protein expression in models of immobility- and denervation-induced muscle atrophy [33,34,35,36]. Studies have also shown that p53 overexpression leads to the emergence of senescent traits and muscle atrophy [33,37], and muscle-specific *p53*-knockout (KO) mice were partially resistant to immobilization-induced skeletal muscle atrophy [33]. Conversely, muscle atrophy was induced in a p53 loss-of-function model created through the overexpression of dominant-negative p53 protein [38]. In an animal model of immobilization, the degree of muscle atrophy was found to be similar between muscle-specific *p53*-KO mice and wild-type mice [36], and aging-related muscle atrophy was not suppressed in the same KO mice described above [39]. Thus, the findings regarding the role of p53 in muscle atrophy have not always been in agreement with each other. The relationship between cisplatin-induced skeletal muscle atrophy and p53 is also yet to be investigated.

In this present study, the treatment of C2C12 myotubes with cisplatin caused an increase in the *Bax/Bcl-2* ratio, p53 and phospho-p53 protein levels, and mRNA expression of multiple p53 target genes. PFT-α reduced the *Bax/Bcl-2* ratio as well as mRNA levels of *PUMA*, *p21*, and muscle-specific ubiquitin ligases (*Atrogin-1* and *MuRF1*). Furthermore, PFT-α reduced the mitochondrial damage (decrease in membrane potential, mitochondrial mass, and ATP production) and increased intracellular ROS production induced by cisplatin. Additionally, the inhibitor restored the cisplatin-induced suppression of the glycolytic system. Each of these findings suggests that cisplatin activates p53 and causes damage and dysfunction in C2C12 myotubes. Mitochondrial damage and ROS overproduction have already been implicated in cisplatin-induced activation of muscle-specific ubiquitin ligases [16]. The present study has revealed that p53 is also involved in the process of cisplatin-induced C2C12 myotube atrophy, and thus its inhibition by PFT-α reduces the activation of muscle degradation.

We have previously reported that MitoQ, similar to PFT-α, reduces cisplatin-induced mitochondrial damage, ROS overproduction, and muscle-specific ubiquitin ligase activation, and also restores the MyHC protein levels in C2C12 myotubes [16]. In this present study, we unexpectedly observed no improvement in the levels of these proteins upon investigating the impact of PFT-α on skeletal muscle-specific proteins (MyHC, muscle-specific actin, and myoglobulin). Thus, although MitoQ and PFT-α are similar with respect to their impacts on cisplatin-induced mitochondrial damage, ROS regulation, and suppression of muscle-specific ubiquitin ligase activation, they have conflicting effects on skeletal muscle-specific protein levels.

In an effort to reveal the reason for this dissimilarity, we investigated muscle-specific protein synthesis in drug-treated C2C12 cells. Cisplatin-induced skeletal muscle atrophy is believed to be associated with not only the activation of muscle-specific ubiquitin ligases, but also reduced levels of IGF1, MyoD1, and myogenin [11,40,41,42,43,44]. Therefore, we compared the impacts of PFT-α and MitoQ on *Igf1*, *MyoD1*, myogenin, and *MyHC* gene expression. On one hand, cisplatin markedly reduced the expression of these genes, whereas MitoQ significantly improved the expression of myogenin and *MyHC* isoforms other than *Myh2*. On the other hand, PFT-α had no effect on the expression of any of these genes other than *Myh7*. These findings suggest that MitoQ improves the levels of muscle-specific proteins not only by suppressing their degradation but also by improving their synthesis, whereas PFT-α does not improve muscle protein synthesis, as evidenced by the absence of an improvement in the muscle-specific protein levels. These observations suggest that cisplatin-induced p53 activation leads to increased muscle protein degradation but is not directly involved in the suppression of muscle protein synthesis. In other words, cisplatin-induced inhibition of muscle protein synthesis seems to occur via a pathway separate from that of p53 activation. The specific mechanisms underlying this pathway are not clear and require further investigation.

Our study shows that the role of cisplatin-activated p53 in muscle atrophy is limited to increasing muscle protein degradation, and that it is not implicated in the suppression of muscle protein synthesis. Previous studies have offered contradictory findings on the role of p53 in skeletal muscle atrophy, and they did not arrive at the same conclusions [33,36,37,38,39]. Based on the findings of this study, one of the reasons for this inconsistency may stem from the varying relative influence of increased muscle protein degradation and suppressed muscle protein synthesis in different experimental systems because of the varying impact of p53 activation or suppression on these two mechanisms underlying muscle atrophy.

The experimental conditions used in this study caused cell atrophy without cell death, as demonstrated in our previous study [16]. Therefore, this study showed that cisplatin causes skeletal muscle atrophy in C2C12 myotubes without causing apoptosis, despite increasing the *PUMA* level and *Bax/Bcl-2* ratio and causing mitochondrial damage and ROS overproduction via p53 activation. Besides cell cycle arrest and apoptosis, p53 activation has also been shown to cause cellular senescence in dividing cells [45]. However, recent evidence has revealed that p53 induces cellular senescence even in non-dividing cells [46]. Indeed, several studies have reported that p53 and p21 cause cellular senescence and atrophy in skeletal muscle cells, resulting in the development of sarcopenia [47,48,49]. These findings suggest that cisplatin-induced myotube atrophy may be a result of cellular senescence caused by p53 activation, although this fact requires further investigation including age-related sarcopenia.

This study possesses several limitations. Firstly, we did not identify the p53-independent pathway responsible for the cisplatin-induced inhibition of muscle-specific protein and gene expression. To address this, further investigation is required to assess the effects of cisplatin and PFT-α on the activities of several transcription factors that bind to skeletal muscle-specific genes (e.g., MyoD, myogenin, NFAT, and MEF2), as well as the regulation of muscle protein synthesis via the Akt-mTOR pathway. Secondly, the direct target of cisplatin involved in p53 activation remains unclear. Lastly, it is important to note that our results were based solely on pharmacological analyses. Therefore, it is important to validate our findings through alternative approaches such as genetic studies. These aspects should be addressed in future studies.

## 4. Materials and Methods

### 4.1. Chemicals

The stocks for cisplatin (479306; Sigma-Aldrich, St. Louis, MO, USA), PFT-α (P4359, Sigma-Aldrich), and MitoQ (HY-100116A, MedChemExpress, Monmouth Junction, NJ, USA) were prepared in dimethylsulfoxide (DMSO). All the solutions were stored at −80 °C.

### 4.2. Cell Culture and Treatment

Mouse C2C12 myoblasts were obtained from KAC Co., Ltd. (EC91031101-F0; Kyoto, Japan). C2C12 myoblast culture and differentiation into myotubes was performed as described previously [16]. Briefly, C2C12 myoblasts were grown in Dulbecco’s modified Eagle’s medium (DMEM; Thermo Fisher Scientific, Waltham, MA, USA) supplemented with 10% fetal bovine serum (JRH Biosciences, Lenexa, KS, USA) and 100 unit/mL penicillin-100 μg/mL streptomycin (Thermo Fisher Scientific) at 37 °C, in a humidified atmosphere containing 5% CO_2_. For differentiation into myotubes, these cells were put into differentiation medium consisting of DMEM supplemented with 2% horse serum (Thermo Fisher Scientific) and cultured for 5 days. Myotubes were then treated with 50 μmol/L cisplatin or vehicle (0.02% DMSO) for 0.5, 2, 6, or 24 h and then subjected to further analysis; PFT-α (20 or 50 μmol/L) [26,27] and MitoQ (0.16 or 0.4 μmol/L) were added 30 min prior to cisplatin treatment.

### 4.3. Western Blotting

A cell lysis buffer containing a protease inhibitor and serine phosphatase inhibitor (10×) (#9803, Cell Signaling Technology, Danvers, MA, USA) was used to isolate proteins from C2C12 myotubes (3.7 × 10^4^ cells/cm^2^) that had been treated with 50 μmol/L cisplatin for 0.5, 2, 6, and 24 h. The p53, phosphorylated p53 (phospho-p53), MyHC, and muscle-specific actin protein levels were measured using a capillary-based Western blotting instrument, Jess Simple Western System (ProteinSimple, San Jose, CA, USA), as previously described [16]. Proteins were denatured at 95 °C for 5 min using reagents provided by the manufacturer and applied to multi-well plates. Denatured proteins were then separated with the 12–230 kDa Jess Separation Module (SM-W004, ProteinSimple) and bound with the antibodies listed below. Each of the bound antibodies were detected using the anti-goat, rabbit, or mouse Detection Modules (DM-006, DM-001, or DM-002, ProteinSimple). Protein separation and detection was performed according to the manufacturer’s instructions by capillary electrophoresis, antibody binding and visualization of HRP conjugates. Next, the primary and secondary antibodies were removed using the RePlex Module (RP-001, ProteinSimple), protein levels of glyceraldehyde 3-phosphate dehydrogenase (GAPDH) were then detected using the same methods as previously reported [16]. Peak areas were automatically calculated using Compass Simple Western software, version 5.0.1 (ProteinSimple), but baselines were adjusted using manual settings and the respective peak areas were corrected for GAPDH peak areas. The antibodies used are as follows: anti-p53 antibody (25 µg/mL; AF1355; R&D Systems, Minneapolis, MN, USA), 50-fold diluted anti-p53 (Ser15) antibody (9284S; Cell Signaling Technology, Danvers, MA, USA), 5 µg/mL anti MyHC antibody (MAB4470; R&D Systems), 10 μg/mL actin (muscle specific) antibody (HHF35; NBP2-34230; Novus Biologicals, LLC, Centennial, CO, USA), and 10 μg/mL GAPDH antibody (AF5718; Novus Biologicals).

### 4.4. Gene Expression

RNA was isolated from C2C12 myotubes (3.7 × 10^4^ cells/cm^2^) treated with 50 µmol/L cisplatin for 2, 6, or 24 h using the RNeasy Mini Kit (74106; QIAGEN, Valencia, CA, USA). Total RNA was reverse transcribed to complementary DNA (cDNA) using the High-Capacity cDNA Reverse Transcription Kit (4368813; Thermo Fisher Scientific). Expression of mRNA was determined with QuantStudio 7 Flex Real-Time PCR System (Thermo Fisher Scientific) using TaqMan Fast Advanced Master Mix (4444557; Thermo Fisher Scientific) and gene-specific TaqMan primers/probes (Thermo Fisher Scientific). The 18S ribosomal RNA gene was used as an internal reference to normalize target gene expression using the ΔΔCt method. The list of primers/probes (Thermo Fisher Scientific) is provided in Supplementary Table S1.

### 4.5. Evaluation of Mitochondrial ATP Production Rate and Glycolysis

C2C12 myotubes (1 × 10^5^ cells/cm^2^) were treated with 50 μmol/L cisplatin for 24 h and then XFp Extracellular Flux Analyzer (Agilent Technologies, Santa Clara, CA, USA) was used to OCR and ECAR of C2C12 myotubes were measured, as described in a previous report [16]. The rate of ATP production from mitochondrial respiration was measured using a Seahorse XFp Real-Time ATP Rate Assay Kit (103591-100; Agilent Technologies). A Seahorse XFp Glycolysis Stress Test Kit (103017-100; Agilent Technologies) was used to evaluate glycolysis. The OCR and ECAR were corrected for protein levels measured with the Pierce BCA Protein Assay (23225; Thermo Fisher Scientific). The data obtained were analyzed with Wave software version 2.6.3 (Agilent Technologies).

(i) Evaluation of ATP production rate from mitochondrial respiration

The ATP production rate of the mitochondria was measured using the Seahorse XFp Real-Time ATP Rate Assay Kit according to the manufacturer’s instructions and protocol. This kit can be used to quantify the rate of ATP production from mitochondrial respiration in living cells. To measure the rate of ATP production from mitochondrial respiration, oligomycin and rotenone/antimycin A mixture were added and the cultures were then monitored for OCR. mitoATP production rate rates were calculated by OCR based on mitochondrial respiration using Wave software (Agilent Technologies).

(ii) Glycolysis evaluation

The Seahorse XFp Glycolysis Stress Test Kit was used to evaluate glycolytic capacity according to the amount of sugar uptake into the cell. ECAR was measured to assess glycolysis. Glucose, the ATP synthesis inhibitor oligomycin and the glycolysis inhibitor 2-deoxy-D-glucose were added according to the manufacturer’s instructions and protocol.

### 4.6. ROS Levels

Intracellular ROS levels in C2C12 myotubes (4.5 × 10^4^ cells/cm^2^) treated with 50 µmol/L cisplatin for 2 or 24 h were measured using 5-(and-6)-chloromethyl-2′,7′-dichlorodihydrofluorescein diacetate, acetyl ester (CM-H_2_DCFDA; C6827; Thermo Fisher Scientific). Briefly, ROS levels were determined by adding 5 μmol/L CM-H_2_DCFDA to the cells for 30 min at 37 °C. The fluorescence intensity was measured using a Flex station 3 multimode microplate reader (Molecular Devices, San Jose, CA, USA) at λ_ex_/λ_em_ = 485/525 nm.

### 4.7. Mitochondrial Mass Measurements and Membrane Potential Assays

Mitochondrial mass and membrane potential were assessed using MitoGreen (PK-CA707-70054; PromoCell, Heidelberg, Germany) and Image-iT tetramethylrhodamine, methyl ester (TMRM; I34361; Thermo Fisher Scientific) reagents, respectively. Briefly, C2C12 myotubes (4.5 × 10^4^ cells/cm^2^) were treated with 50 μmol/L cisplatin for 24 h and then a total of 50 nmol/L MitoGreen and 100 nmol/L TMRM were added to the cells, respectively. The cells were incubated for 30 min at 37 °C. Fluorescence was measured using a Flex station 3 multimode microplate reader at λ_ex_/λ_em_ = 485/525 nm and λ_ex_/λ_em_ = 544/590 nm, respectively.

### 4.8. Myoglobin Levels

Intracellular myoglobin levels in C2C12 myotubes (4.5 × 10^4^ cells/cm^2^) treated with 50 µmol/L cisplatin for 24 h were measured using the Myoglobin SimpleStep ELISA Kit (ab210965; Abcam, Cambridge, UK) following the manufacturer’s manual. The absorbance at 450 nm was measured with an Infinite 200 PRO microplate reader (Tecan, Grödig, Austria).

### 4.9. Statistical Analysis

Data were presented as mean ± standard deviation (SD). The results were analyzed using GraphPad Prism version 7.04 (GraphPad Software Inc., San Diego, CA, USA). Student’s or Welch’s *t* test was performed for comparisons among two groups. Dunnett’s or Tukey’s test was used for three or four group comparisons, respectively. In the two-way analysis of variance (ANOVA) test, if a significant difference was observed in the cisplatin × PFT-α (or MitoQ) interaction, an all-group comparison (Tukey’s test) was performed; if there was no significant difference, the Dunnett’s test was performed separately for cells with and without cisplatin. In addition, *p* < 0.05 indicated statistically significant differences.

## 5. Conclusions

Using a cell model of cisplatin-induced C2C12 myotubes atrophy, we showed that p53 activation is directly implicated in cisplatin-induced increase in the *Bax/Bcl-2* ratio, mitochondrial damage, ROS production, and activation of atrogenes, but has no impact on muscle protein synthesis. The role of p53 in muscle atrophy differs between the processes of muscle protein degradation and synthesis, and these mechanism-dependent differences may be one of the reasons why previous studies have failed to arrive at a consensus regarding the role of p53 in skeletal muscle atrophy.

## Data Availability

Data are available upon reasonable request from the corresponding author.

## Supplementary Materials

The following supporting information can be downloaded at: https://www.mdpi.com/article/10.3390/ijms24119176/s1.
