# Supplementary material for: Role of p53 in Cisplatin-Induced Myotube Atrophy
Source: Int J Mol Sci. 2023 May 24;24(11):9176. doi: 10.3390/ijms24119176 (PMC10253206; doi:10.3390/ijms24119176)
Supplement: Supplementary file 1 [file ijms-24-09176-s001.zip › Supplementary_Fig_Table.pdf]

Role of p53 in Cisplatin-induced Myotube Atrophy

Chinami Matsumoto, Hitomi Sekine, Nana Zhang, Sachiko Mogami, Naoki Fujitsuka, and Hiroshi Takeda

Supplementary Information

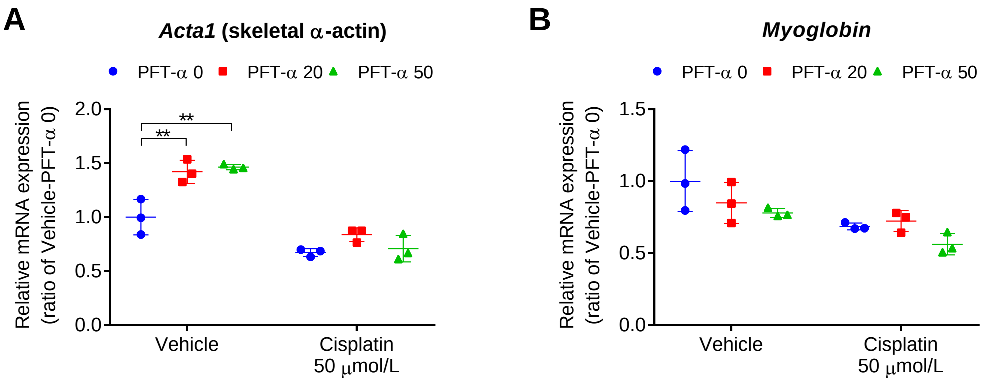

Supplementary Figure 1. Effect of PFT-α on the expression of skeletal α-actin and myoglobin genes in C2C12 myotubes.

(**A**, **B**) mRNA expression levels in cisplatin-treated C2C12 myotubes with or without PFT-α (20 or 50 μmol/L). *Acta1* (skeletal α-actin)(**A**); two-way ANOVA revealed a significant effect of cisplatin ($p < 0.01$) and cisplatin × PFT-α ($p < 0.01$). **$p < 0.01$, Tukey's multiple-comparisons post hoc test. *Myoglobin* (**B**); two-way ANOVA revealed a significant effect of cisplatin ($p <$ 0.01). The values are expressed as the mean ± SD ($n = 3$ in each group).

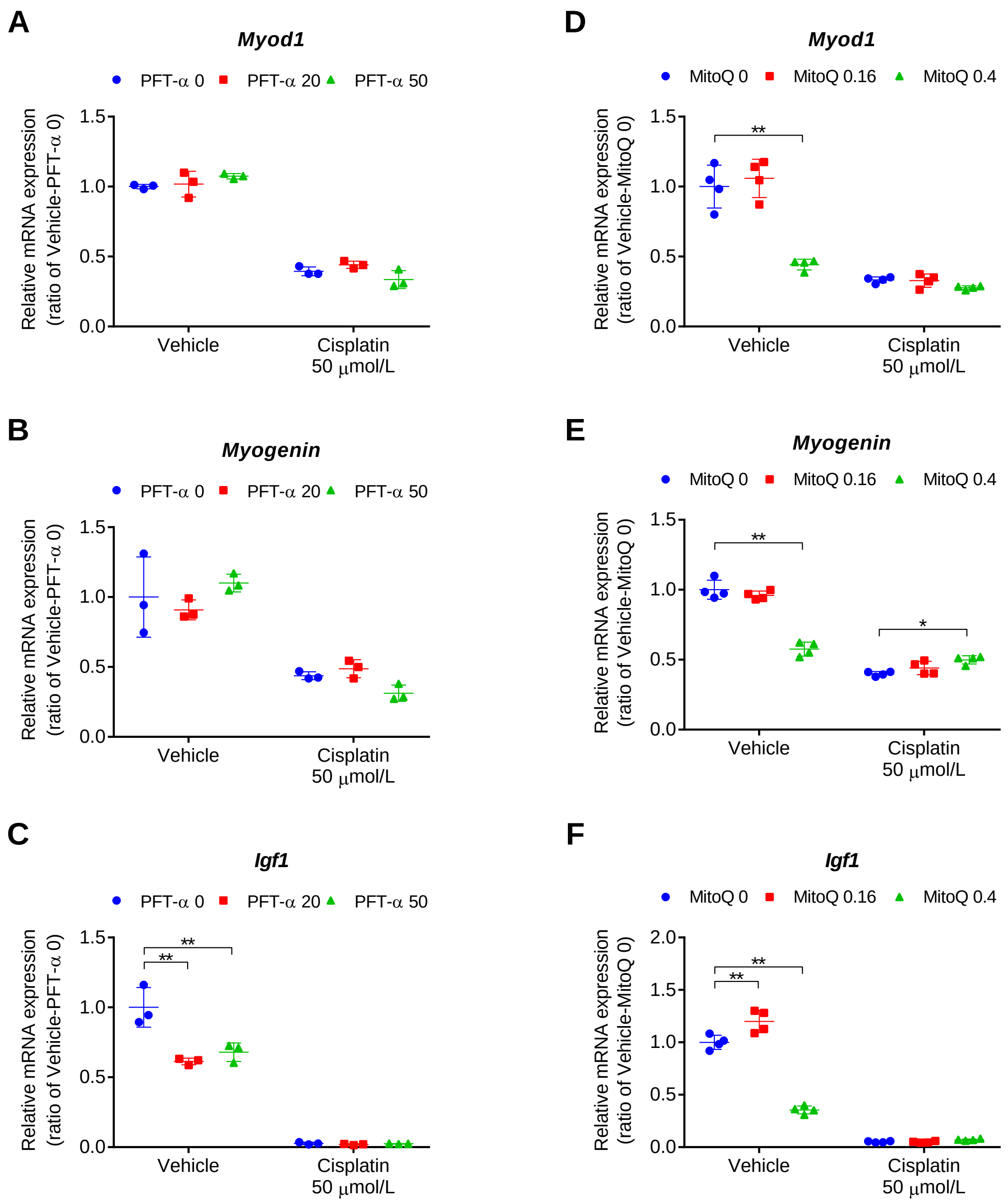

Supplementary Figure 2. Effect of PFT-*α* on the gene expression of the regulators of muscle differentiation in C2C12 myotubes.

Evaluation of the mRNA expression levels in cisplatin-treated C2C12 myotubes treated with 20 or 50 μmol/L PFT-*α* (**A**–**C**) or 0.16 or 0.4 μmol/L MitoQ (**D**–**F**). *Myod1* (**A**, **D**), *Igf1* (**C**, **F**), *Myogenin* (**E**); two-way ANOVA revealed a significant effect of cisplatin ($p < 0.01$) and cisplatin × PFT-*α* or MitoQ ($p < 0.05$). *$p < 0.05$, **$p < 0.01$, Tukey's multiple-comparisons post hoc test. *Myogenin* (**B**); two-way ANOVA revealed a significant effect of cisplatin ($p < 0.01$). The values are expressed as the mean ± SD (n = 3–4 in each group).

Supplementary Table 1. Primers used for RT-PCR

Target	Gene name	Assay ID
Rn18s	18S ribosomal RNA	Mm04277571_S1
PUMA (Bbc3)	BCL2 binding component 3	Mm00519268_m1
p21 (Cdkn1a)	cyclin-dependent kinase inhibitor 1A (P21)	Mm00432448_m1
Tigar	Trp53 induced glycolysis regulatory phosphatase	Mm00621530_m1
Bax	BCL2-associated X protein	Mm00432051_m1
Bcl-2	B cell leukemia/lymphoma 2	Mm00477631_m1
Foxo4	forkhead box O4	Mm00840140_g1
Atrogin-1 (Fbxo32)	F-box protein 32	Mm00499523_m1
MuRF1 (Trim63)	tripartite motif-containing 63	Mm01185221_m1
Myh7	myosin, heavy polypeptide 7	Mm00600555_m1
Myh2	myosin, heavy polypeptide 2	Mm01332564_m1
Myh1	myosin, heavy polypeptide 1	Mm01332500_gH
Myh4	myosin, heavy polypeptide 4	Mm01332541_m1
Acta1	actin, alpha 1, skeletal muscle	Mm00808218_g1
Mb	myoglobin	Mm00442968_m1
Myod1	myogenic differentiation 1	Mm00440387_m1
Myog	myogenin	Mm00446194_m1
Igf1	insulin-like growth factor 1	Mm00439560_m1
Rn18s	18S ribosomal RNA	Mm04277571_S1
